# Olfactory dysfunction as a marker of neurodegenerative disease in older adults

**DOI:** 10.1007/s40520-026-03404-5

**Published:** 2026-04-30

**Authors:** Kateřina Klíčová, Kateřina Menšíková, Dorota Šebelová, Petra Polčáková, Michal Kaleta, Sarah Elizabeth Victoria Cook, Zuzana Ildžová, Lenka Satke, Dalibor Zimek, Petr Kaňovský

**Affiliations:** 1 Department of Neurology, Faculty of Medicine and Dentistry, Palacky University, Olomouc, Czechia; 2 Department of Neurology, University Hospital Olomouc, Olomouc, Czechia; 3 Laboratory of Growth Regulators, Institute of Experimental Botany, Czech Academy of Sciences & Palacky University, Olomouc, Czechia

**Keywords:** Olfactory dysfunction, Older adults, Geriatric assessment, Neurodegenerative disease, Sniffin’ Sticks

## Abstract

**Background:**

Early identification of neurodegenerative disorders in older adults remains a major challenge in geriatric clinical practice. Olfactory dysfunction is a common feature of several neurodegenerative disorders, particularly synucleinopathies, where it often precedes motor and cognitive symptoms, whereas in primary tauopathies olfactory function is usually preserved or only mildly impaired.

**Aims:**

This study aimed to evaluate the diagnostic utility of the 12-item Sniffin’ Sticks Test (SST-12) for identifying neurodegenerative disease in older adults.

**Methods:**

A total of 120 individuals were included: 72 with synucleinopathies, 23 with tauopathies, and 25 healthy controls. Olfactory function was assessed using the SST-12. Group differences were analyzed using ANOVA, Fisher’s exact test, and post hoc procedures. Diagnostic accuracy was evaluated by ROC analysis.

**Results:**

Anosmia occurred in 58.3% of synucleinopathy patients and 65.2% of tauopathy patients but was absent in controls (*p < 0.0001*). Women performed better on several items, while an age effect was observed only for odor T5 (banana) (*p = 0.011*). Subjective olfactory complaints had limited diagnostic value. The full SST-12 showed good accuracy in distinguishing patients from controls (AUC = 0.818; 95% CI 0.744–0.891). A shortened nine-odor version achieved slightly higher accuracy (AUC = 0.844; 95% CI 0.777–0.912). No significant difference was observed between synucleinopathies and tauopathies, including after adjustment for age and sex.

**Discussion:**

These findings support the clinical usefulness of brief olfactory testing in older adults and highlight the potential of selected odor subsets to improve screening efficiency.

**Conclusion:**

The SST-12 is a sensitive and practical tool for detecting olfactory dysfunction in neurodegenerative disorders. Brief olfactory screening may represent a useful addition to routine geriatric assessment.

## Introduction

The human olfactory system is highly sensitive and specific, capable of detecting tens of thousands of different chemical compounds, often at extremely low concentrations. It is composed of the olfactory epithelium, the olfactory pathway, and central brain structures, which enable the detection of trace amounts of odors, their processing, and integration with emotions and memory. Olfactory disorders occur in a variety of neurological conditions. In recent years, interest in their association with neurodegenerative diseases has increased. Olfactory dysfunction, typically defined as a reduced ability to detect or identify odors, often precedes the manifestation of other clinical symptoms [1–5].

Many individuals with olfactory impairment are unaware of their deficit, which is often detected only through objective testing. Although olfactory function naturally declines with age and its weakening is a common feature of aging, numerous studies have shown that patients with Parkinson’s disease (PD) and Alzheimer’s disease (AD) exhibit impairments in odor detection, discrimination, and identification compared with age-matched healthy controls.

 [1, 2]. Olfactory dysfunction is one of the most common non-motor symptoms of PD. Postmortem studies confirm a substantial loss of neurons in the olfactory bulb and related regions [6]. A mere anamnesis of olfactory impairment is insufficient, as subjective assessment of smell is generally unreliable—patients often fail to notice its decline or loss [7–10]. Olfactory testing may therefore represent a sensitive tool for the early detection of neurodegenerative disease.

Based on the predominant pathological protein, neurodegenerative diseases can be classified into synucleinopathies (idiopathic Parkinson’s disease – iPD, dementia with Lewy bodies – DLB, multiple system atrophy – MSA) and tauopathies (progressive supranuclear palsy – PSP, corticobasal degeneration – CBD, and certain forms of frontotemporal dementia – FTD) [11].

Olfactory dysfunction is a characteristic early symptom of synucleinopathies, particularly iPD and DLB, where it occurs in the majority of patients and often precedes motor or cognitive symptoms [12, 13]. In MSA, olfactory function is usually preserved, although some studies have reported a mild decrease in sensitivity in a subset of patients. In contrast, in primary 4R-tauopathies—PSP and CBD—olfactory function is usually preserved or only mildly impaired compared with synucleinopathies [14].

Various methods are used to assess olfactory function, including the electro-olfactogram, which measures electrical activity at the surface of the olfactory epithelium for olfactory event-related potentialsthe Sniff Magnitude Test, a reflex-based method with good reliability [15], and functional magnetic resonance imaging, which, however, is financially and technically demanding. Other approaches include commercially available psychophysical tests of olfactory abilities (e.g., UPSIT, Sniffin’ Sticks), which are less time- and cost-intensive and suitable for routine clinical practice. Normative data for the Sniffin’ Sticks (TDI score) were published in the study by Hummel et al. (2007) [16], which involved more than 3,000 individuals and was later supplemented by an update comprising over 9,000 subjects [17]. In our study, we used the screening version of the Sniffin’ Sticks with 12 odors (Screening 12 Test CZ LA-13-00001, MediSense), which enables a rapid and practical assessment of olfactory function. In geriatric practice, a rapid, low-cost screening tool capable of identifying older adults at increased risk of neurodegenerative disease would be of clinical value.

## Methods

Patients were recruited through the Department of Neurology, Palacky University Olomouc and University Hospital Olomouc, Czech Republic. Informed consent was obtained from all participants involved in the study. The project was approved by the Ethics Committee of the University Hospital Olomouc and the Faculty of Medicine and Dentistry, Palacky University Olomouc.

Patients with the following clinical diagnoses were included in the study: iPD, DLB, MSA, PSP, CBS, and FTD. Based on clinical diagnosis and detailed phenotypic presentation, participants were divided into two main groups synucleinopathies and tauopathies. The synucleinopathy group included patients with idiopathic Parkinson’s disease (iPD), dementia with Lewy bodies (DLB), and multiple system atrophy (MSA), while the tauopathy group included patients with progressive supranuclear palsy (PSP), corticobasal syndrome (CBS), and frontotemporal dementia (FTD).

In total, 120 participants were enrolled in the study: 72 patients with synucleinopathies (iPD and MSA, DLB), 23 patients with tauopathies (PSP, FTD, CBS), and 25 individuals in the control group. The clinical diagnoses were established according to current validated international clinical diagnostic criteria for the respective neurodegenerative disorders [18–21]. The control group consisted of individuals with neurological complaints unrelated to neurodegenerative disorders (e.g., lumbar radiculopathy, headache, back pain). All patients underwent comprehensive clinical and paraclinical examinations in accordance with the currently used and validated diagnostic criteria for the respective neurodegenerative disorders.

The diagnosis was always established based on a combination of the clinical presentation and the results of supportive investigations, when indicated.

Olfactory function was assessed using the Sniffin’ Sticks Screening 12 Test (SST-12) (Screening 12 Test CZ LA-13-00001, MediSense), a screening tool comprising 12 common odors designed for basic evaluation of olfactory performance. Participants selected the correct answer from four alternatives. Based on the total number of correct responses, subjects were classified into categories of anosmia, hyposmia, or normosmia. Subjective olfactory impairment was recorded as a dichotomous variable (yes/no). The validity and application of this screening test have been described, for example, in the study by Pinkhardt et al. (2019) [22]. Thresholds for the SST-12 were defined as normosmia (SST-12 ≥ 11), hyposmia (10 > SST-12 > 6), or anosmia (SST-12 ≤ 6) [23, 24].

Cognitive performance was assessed using the Mini-Mental State Examination (MMSE). In addition, demographic and clinical data were recorded, including age, sex, and smoking status.

Statistical analyses were performed using IBM SPSS Statistics for Windows, version 23.0 (IBM Corp., Armonk, NY, USA). Fisher’s exact test and analysis of adjusted residuals were used to compare categorical variables. Differences in quantitative variables between groups were assessed using ANOVA (with Bonferroni post hoc test) or nonparametric tests (Mann–Whitney U, Kruskal–Wallis ANOVA), depending on the data distribution. The diagnostic performance of the olfactory test was evaluated using ROC analysis (determination of AUC and optimal cut-off value).

To assess whether differences in olfactory performance between diagnostic groups persisted after adjustment for potential confounders, an analysis of covariance (ANCOVA) was performed with the SST-12 total score as the dependent variable, diagnostic group as a fixed factor, and age and sex as covariates. Post hoc pairwise comparisons were adjusted using the Bonferroni correction.

## Results

### Demographic characteristics

A total of 120 individuals were included in the analysis: 72 with synucleinopathies (iPD, DLB, MSA), 23 with tauopathies (PSP, CBD, FTD), and 25 healthy controls. The distribution of sex did not differ significantly between groups (female/male: 38/34 in synucleinopathies, 10/13 in tauopathies, 13/12 in controls; *p* = 0.739).

The age distribution within each group followed a normal distribution, allowing the use of parametric analysis. ANOVA revealed differences in mean age between groups (*p = 0.005*); post hoc Bonferroni testing showed that patients with synucleinopathies were younger than those with tauopathies (*p = 0.004*). Differences between the other groups were not statistically significant (*p > 0.05*). Age (mean ± SD, range): synucleinopathies 65.13 ± 8.66 (44–82), tauopathies 72.13 ± 7.56 (55–82), controls 67.56 ± 10.14 (46–82). The fact that patients with synucleinopathies were younger likely reflects the earlier onset of symptoms characteristic of these disorders compared with tauopathies, which typically manifest at an older age.

### Olfactory tests

The results of the olfactory tests (T1–T12) revealed significant group differences in nine out of twelve odors tested (T1, T3, T4, T5, T7, T8, T9, T11, and T12) (Table 1). The largest differences were observed between patients and controls, with controls showing substantially higher success rates in odor identification.

Overall classification based on the number of correct responses confirmed different distributions of anosmia, hyposmia, and normosmia among the groups (*p < 0.0001*). Anosmia was present in 58.3% of patients with synucleinopathies, 65.2% of patients with tauopathies, and in none of the controls. Hyposmia was most frequent in controls (64%), whereas patients more often exhibited severe impairment (anosmia). This finding reflects the fact that patients with neurodegenerative disease typically lose olfactory function completely, while healthy individuals more often present with only a pertially reduced olfactory capacity. The success rates achieved in identifying individual odors are presented below.

- **T1 (orange)**: patients with synucleinopathies and tauopathies achieved lower success rates than controls (*p = 0.009*).
- **T2 (leather)**: differences between groups were not significant (*p = 0.542*). (It should be noted, however, that in Czech the term *“kůže”* is ambiguous—it may refer either to human skin or to leather material, which could have contributed to difficulties in identification)
- **T3 (cinnamon)**: success rates were significantly lower in patients compared with controls (*p = 0.001*).
- **T4 (peppermint)**: patients performed worse than controls (*p < 0.0001*).
- **T5 (banana)**: controls recognised the odor more frequently than patients (*p < 0.0001*).
- **T6 (lemon)**: differences were not significant (*p = 0.478*).
- **T7 (licorice)**: patients showed lower success rates (*p = 0.049*).
- **T8 (coffee)**: controls performed significantly better than patients (*p = 0.001*).
- **T9 (clove)**: differences were significant, with patients achieving lower success rates (*p = 0.039*).
- **T10 (pineapple)**: differences were not significant (*p = 0.439*).
- **T11 (rose)**: controls had higher success rates in odor identification (*p = 0.012*).
- **T12 (fish)**: success rates were significantly lower in patients compared with controls (*p = 0.001*).

**Fig. 1:**
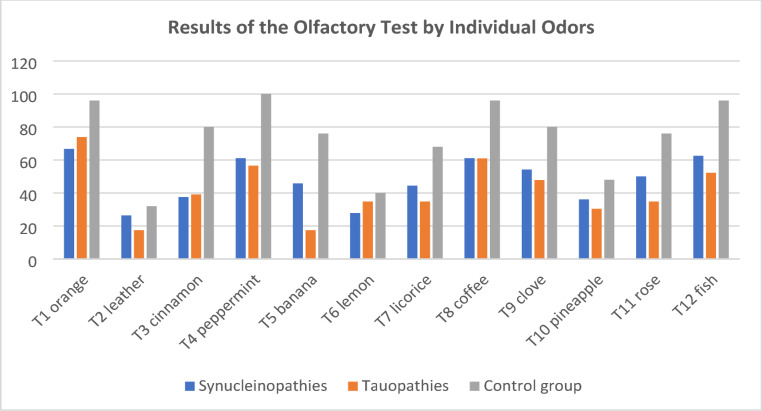
Graphical Representation of Olfactory Test Results by Individual Odors (T1–T12)

The data are summarised in Table 1 and illustrated in Fig. 1.

Overall, the odors with the greatest discriminatory potential were orange, cinnamon, peppermint, banana, licorice, coffee, clove, rose, and fish, which were almost always correctly identified by controls but frequently failed in patients with neurodegenerative disease (Table 1; Fig. 1).

No significant difference in SST-12 scores was observed between patients with synucleinopathies and tauopathies (*p = 0.223*). Similar results were obtained for the shortened version of the test (*p = 0.278*).

To further assess whether group differences in olfactory performance persisted after adjustment for potential confounders, an ANCOVA was performed with SST-12 total score as the dependent variable, diagnostic group as a fixed factor, and age and sex as covariates. Diagnostic group remained a significant factor, F(2,115) = 17.236, *p* < 0.001, partial η² = 0.231. Post hoc comparisons with Bonferroni correction showed that both synucleinopathies and tauopathies differed significantly from controls (both *p* < 0.001), whereas no significant difference was observed between synucleinopathies and tauopathies (*p = 1.000*). Age (*p = 0.001*) and sex (*p < 0.001*) also had significant effects on olfactory performance.

**Table 1:** Olfactory Identification Ability for Individual Odors (T1–T12) Across Cohorts

		Synucleinopathies		Tauopathies		Control group		*p*
		*n*	%	*n*	%	*n*	%	
**T1** **orange**	1	**48**	**66.70**	17	73.90	**24**	**96.00**	**0.009**
	0	**24**	**33.30**	6	26.10	**1**	**4.00**	
**T2** **leather**	1	19	26.40	4	17.40	8	32.00	0.542
	0	53	73.60	19	82.60	17	68.00	
**T3** **cinnamon**	1	**27**	**37.50**	9	39.10	**20**	**80.00**	**0.001**
	0	**45**	**62.50**	14	60.90	**5**	**20.00**	
**T4** **peppermint**	1	**44**	**61.10**	13	56.50	**25**	**100.00**	**< 0.0001**
	0	**28**	**38.90**	10	43.50	**0**	**0.00**	
**T5** **banana**	1	33	45.80	**4**	**17.40**	**19**	**76.00**	**< 0.0001**
	0	39	54.20	**19**	**82.60**	**6**	**24.00**	
**T6** **lemon**	1	20	27.80	8	34.80	10	40.00	0.478
	0	52	72.20	15	65.20	15	60.00	
**T7** **licorice**	1	32	44.40	8	34.80	**17**	**68.00**	**0.049**
	0	40	55.60	15	65.20	**8**	**32.00**	
**T8** **coffee**	1	**44**	**61.10**	14	60.90	**24**	**96.00**	**0.001**
	0	**28**	**38.90**	9	39.10	**1**	**4.00**	
**T9** **clove**	1	39	54.20	11	47.80	**20**	**80.00**	**0.039**
	0	33	45.80	12	52.20	**5**	**20.00**	
**T10** **pineapple**	1	26	36.10	7	30.40	12	48.00	0.439
	0	46	63.90	16	69.60	13	52.00	
**T11** **rose**	1	36	50.00	8	34.80	**19**	**76.00**	**0.012**
	0	36	50.00	15	65.20	**6**	**24.00**	
**T12** **fish**	1	45	62.50	12	52.20	**24**	**96.00**	**0.001**
	0	27	37.50	11	47.80	**1**	**4.00**	
**Result**	**ANOSMIA**	**42**	**58.30**	15	65.20	**0**	**0.00**	**< 0.0001**
	**HYPOSMIA**	21	29.20	6	26.10	**16**	**64.00**	
	**NORMOSMIA**	9	12.50	2	8.70	**9**	**36.00**	

1 – correct responses; 0 – incorrect responses

The groups differed in the results of items T1, T3, T4, T5, T7, T8, T9, T11, and T12. Significant differences were also observed in the overall outcomes (anosmia, hyposmia, normosmia). Statistically significant deviations from the expected frequencies are shown in bold

### Effect of age on olfactory test results

**Fig. 2:**
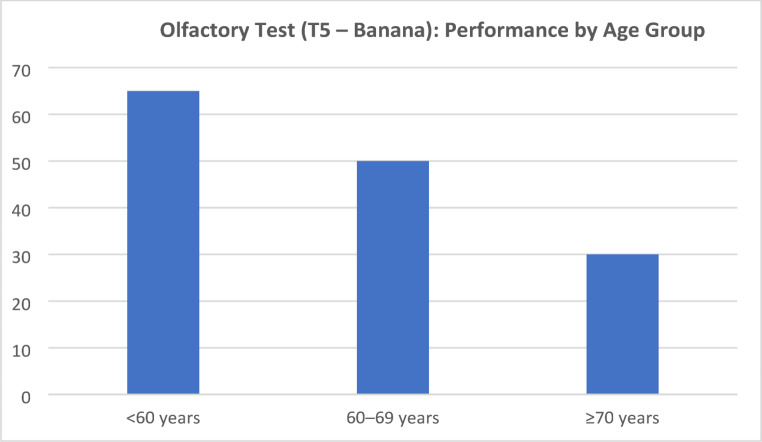
Effect of Age on Olfactory Test Results

An age-related effect was observed in item T5 (banana), where older participants achieved lower success rates (*p = 0.011*). The data are summarized in Table 2 and illustrated in Figure 2.

**Table 2:** Effect of Age on Olfactory Test Results

		Age						*p*
		< 60 years		60–69 years		≥ 70 years		
		*n*	%	*n*	%	*n*	%	
**T1** **orange**	1	16	76.2	40	85.1	33	63.5	0.052
	0	5	23.8	7	14.9	19	36.5	
**T2** **leather**	1	5	23.8	12	25.5	14	26.9	1.000
	0	16	76.2	35	74.5	38	73.1	
**T3** **cinnamon**	1	13	61.9	19	40.4	24	46.2	0.259
	0	8	38.1	28	59.6	28	53.8	
**T4** **peppermint**	1	18	85.7	31	66.0	33	63.5	0.163
	0	3	14.3	16	34.0	19	36.5	
**T5** **banana**	1	16	76.2	20	42.6	20	38.5	**0.011**
	0	5	23.8	27	57.4	32	61.5	
**T6** **lemon**	1	11	52.4	12	25.5	15	28.8	0.084
	0	10	47.6	35	74.5	37	71.2	
**T7** **licorice**	1	13	61.9	24	51.1	20	38.5	0.175
	0	8	38.1	23	48.9	32	61.5	
**T8** **coffee**	1	17	81.0	30	63.8	35	67.3	0.391
	0	4	19.0	17	36.2	17	32.7	
**T9** **clove**	1	15	71.4	25	53.2	30	57.7	0.369
	0	6	28.6	22	46.8	22	42.3	
**T10** **pineapple**	1	9	42.9	17	36.2	19	36.5	0.880
	0	12	57.1	30	63.8	33	63.5	
**T11** **rose**	1	13	61.9	23	48.9	27	51.9	0.637
	0	8	38.1	24	51.1	25	48.1	
**T12** **fish**	1	15	71.4	34	72.3	32	61.5	0.486
	0	6	28.6	13	27.7	20	38.5	

1 – correct responses; 0 – incorrect responses

A statistically significant age-related effect was demonstrated only for test T5. With increasing age, the proportion of correct responses decreased

### Effect of sex on olfactory test results

Analysis by sex showed that women performed better in items T3 (cinnamon), T8 (coffee), and T12 (fish) (*p < 0.05*).

### Subjective olfactory complaints and smoking

Subjective olfactory complaints were reported by 23.6% of patients with synucleinopathies, whereas none were recorded in patients with tauopathies or in controls.

(*p = 0.001*). The presence of subjective complaints was more common in patients with objective anosmia (22,8%) than in those with hyposmia (7%) or normosmia (5%). This difference reached statistical significance (Fisher’s exact test, *p* = 0.040).

Smoking status did not differ significantly between groups (5.6% in synucleinopathies, 8.7% in tauopathies, and 8.0% in controls; Fisher’s exact test, *p* = 0.833). Moreover, smoking was not associated with olfactory test results (*p = 1.000*).

### Cognitive tests (MMSE)

Patients with synucleinopathies achieved higher MMSE scores than patients with tauopathies (28.0 ± 2.3 vs. 24.5 ± 5.4; *p* < 0.0001). Differences between groups defined by olfactory test results (anosmia, hyposmia, normosmia) were not statistically significant.

(*p* = 0.858). No correlation was found between disease duration and MMSE (*p = 0.435*).

### Disease duration

The median disease duration was 36 months in patients with anosmia, 36 months in patients with hyposmia, and 24 months in patients with normosmia. The Kruskal–Wallis ANOVA test revealed no statistically significant differences between these groups (*p = 0.967*).

### ROC analysis

**Fig. 3:**
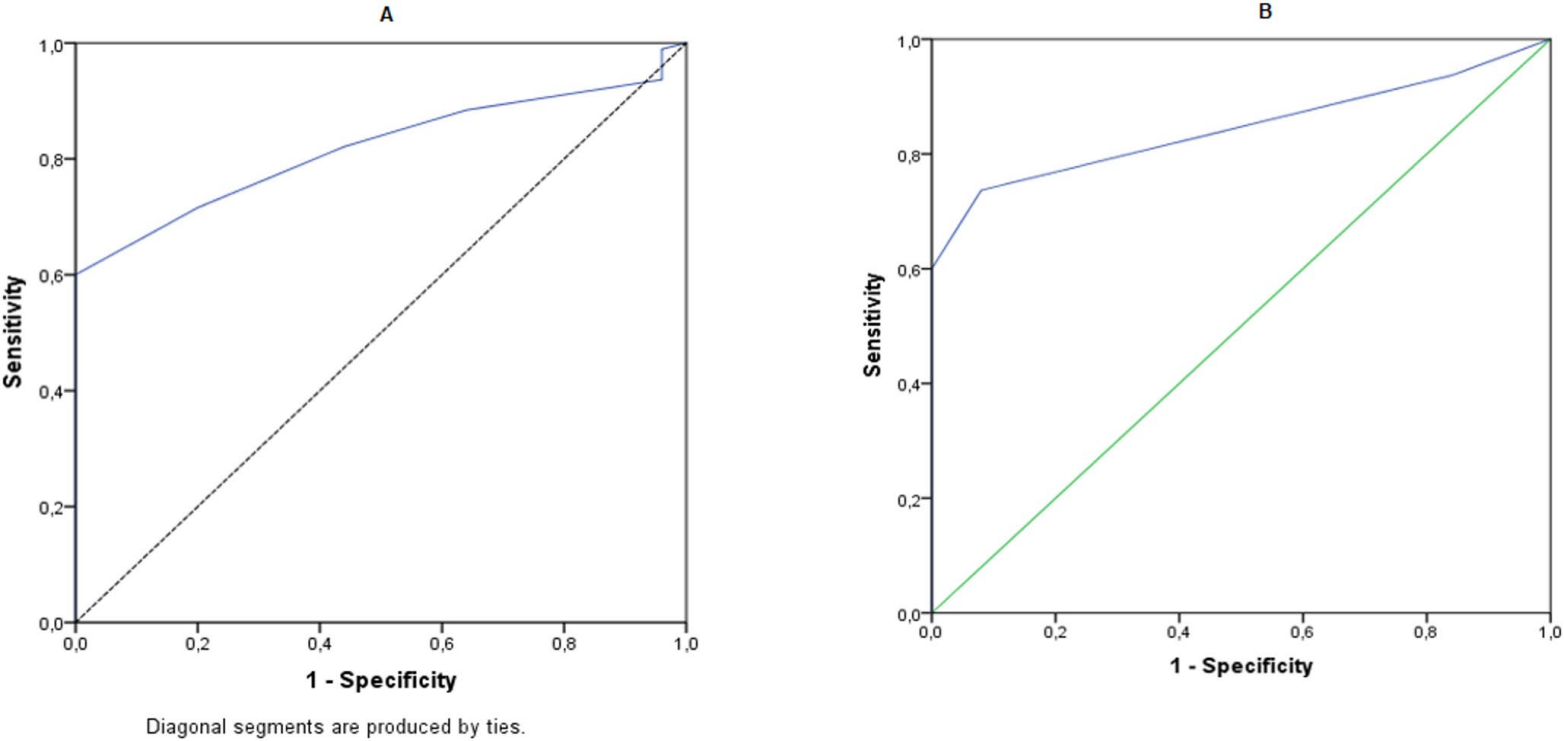
ROC curves for distinguishing patients with synucleinopathies and tauopathies from controls based on the olfactory test in the full (**A**) and reduced (**B**) versions. The curves illustrate the discriminatory ability of both test versions in differentiating between groups

ROC analysis showed that the total number of correct responses in the full 12-item version of the test significantly distinguished between patients and controls (AUC = 0.818; 95% CI: 0.744–0.891; *p* < 0.0001), indicating good discriminatory ability of the test. The optimal cut-off value was 6.5 correct responses (sensitivity = 0.60; specificity = 1.00). The shortened version of the test, including only the odors with the highest discriminatory potential (T1, T3, T4, T5, T7, T8, T9, T11, T12), achieved comparable and even slightly higher diagnostic accuracy (AUC = 0.844; 95% CI: 0.777–0.912; *p* < 0.0001). The optimal cut-off value was 6.5 correct responses (sensitivity = 0.737; specificity = 0.920). See Fig. 3.

## Discussion

Our study confirmed that olfactory dysfunction represents a sensitive marker of neurodegenerative diseases with a good ability to distinguish patients from healthy controls. Using the SST-12, we found that more than half of the patients with synucleinopathies (58.3%) and tauopathies (65.2%) presented with anosmia, whereas none of the controls was anosmic. The full version of the SST-12 achieved good diagnostic accuracy (AUC = 0.818) with an optimal cut-off value of 6.5 correct responses (sensitivity = 0.60; specificity = 1.00). While the AUC values indicate good discriminative ability, the relatively lower sensitivity of the full version suggests that the SST-12 may be more suitable as a screening tool. In our cohort, synucleinopathies and tauopathies could not be distinguished using SST-12. This is consistent with our statistical analysis, including ANCOVA adjusted for age and sex, which confirmed that both patient groups differed significantly from controls, whereas no significant difference was observed between synucleinopathies and tauopathies. These findings suggest that SST-12 may be more suitable for distinguishing individuals with neurodegenerative disease from controls rather than for differentiating between specific neurodegenerative subtypes. Contrary to the established assumption that olfactory dysfunction is more pronounced in synucleinopathies than in tauopathies, we observed a similarly high prevalence of anosmia in both groups. This unexpected finding may be explained by heterogeneity within the tauopathy group, differences in disease stage, and the limited sensitivity of the SST-12 to detect subtle differences between neurodegenerative subtypes. The unequal group sizes may also have reduced the statistical power to detect differences between synucleinopathies and tauopathies.

The odors with the highest discriminatory potential were orange, cinnamon, peppermint, banana, licorice, coffee, clove, rose, and fish, which were almost always correctly identified by the control group but frequently misidentified by the patients. ROC analysis of the shortened version of the test based on these odors confirmed higher diagnostic accuracy (AUC = 0.844), with good sensitivity (0.737) and specificity (0.920). These findings suggest that even a shorter version of the test may be sufficiently reliable and efficient, opening the possibility of developing a practical abbreviated screening set suitable for routine use. Our findings, in line with recent studies, suggest that carefully selected smaller sets of odors may have diagnostic value comparable to longer tests, provided they are validated in the respective population. However, as the shortened version was derived within the same dataset, external validation in independent cohorts is required to confirm these findings.

In our cohort, the item “leather” (T2) showed low discriminatory value, which may be explained by the linguistic ambiguity of the Czech term ,,*kůže*‘‘, as it may refer either to human skin or to leather as a material, potentially affecting odor identification performance. Similar challenges related to cultural and linguistic adaptation of odor items have also been reported in other validation studies [22, 25–28].

### Subjective complaints versus objective testing

Subjective olfactory complaints were reported predominantly by patients with synucleinopathies (23.6%), whereas patients with tauopathies and controls did not report such difficulties. However, even in this group, the agreement between subjective perception and objective test results was low. The presence of complaints was associated only with objective anosmia, while patients with hyposmia did not report problems. This is consistent with studies showing that subjective assessment of olfaction has limited diagnostic value and should not replace standardized testing [23, 29, 30].

### Effect of sex, age, and smoking

Analysis by sex showed that women achieved better results for odors T3 (cinnamon), T8 (coffee), and T12 (fish). This finding is consistent with meta-analyses confirming superior olfactory abilities in women compared with men [31, 32]. The effect of age was limited—we found a significant decline in success rates only for the banana odor (T5), where older participants performed worse. Although olfactory function decreases with age, the impact of neurodegeneration usually outweighs the physiological changes associated with aging [4, 33, 34]. Smoking was not associated with the diagnostic group or with the results of the olfactory test in our cohort. Sharer et al. (2015) [35] reported that in patients with PD who were current smokers, no decline in olfactory function was observed, in contrast to the decrease typically associated with smoking in control subjects.

### Relationship to cognitive performance

In our cohort, patients with synucleinopathies achieved higher MMSE scores than those with tauopathies, reflecting the different cognitive profiles of these disorders. However, the olfactory test results themselves did not correlate with cognitive performance, nor was any association found between disease duration and MMSE scores. Olfactory dysfunction is often described as a prodromal symptom that may precede both cognitive and motor manifestations of certain neurodegenerative diseases, particularly PD and AD [33], and may represent a relatively independent marker. Current evidence suggests that olfactory impairment may predict future cognitive decline, particularly when combined with other biomarkers [36]. Recent findings indicate that patients with PD who exhibited both olfactory dysfunction and amyloid positivity (defined by the cerebrospinal fluid Aβ42/Aβ40 ratio) showed faster deterioration in a range of cognitive and motor measures; moreover, both characteristics were independently associated with an increased risk of cognitive decline and the development of dementia [37].

### Strengths and limitations of the study and clinical applicability

A major strength of our study is the relatively large cohort of patients with two distinct types of neurodegenerative pathology. An additional advantage is the detailed analysis of individual test items and the evaluation of the effects of sex, age, subjective olfactory assessment, and cognitive function. Limitations include the smaller number of patients with tauopathies, the absence of longitudinal follow-up, and the linguistic ambiguity of odor T2 (leather), which may have influenced the results. Nevertheless, our findings support the practical applicability of olfactory testing as a screening tool.

In addition, the inclusion of heterogeneous conditions within the synucleinopathy and tauopathy groups (e.g., MSA with iPD, and FTD with PSP/CBS) may have obscured disease-specific patterns of olfactory dysfunction. Future studies with more homogeneous diagnostic subgroups are needed to better characterize disease-specific olfactory profiles.

In addition MMSE was not included in the multivariable model, as it was not assessed in the control group, which limited its use as a covariate.

Our results confirm the importance of olfactory testing as a reliable tool in older adults. Several specific odors demonstrated the highest discriminatory potential and may serve as the basis for a shortened version. The abbreviated olfactory test represents a rapid and cost-effective tool suitable for clinical practice. The SST-12 may be particularly useful in geriatric and memory clinic settings and could represent a practical addition to routine geriatric assessment.

## Data Availability

All data relevant to the study are included in the article. Additional data are available upon reasonable request to the corresponding author.
